# Predictive model for long COVID in children 3 months after a SARS-CoV-2 PCR test

**DOI:** 10.1186/s12916-022-02664-y

**Published:** 2022-11-30

**Authors:** Manjula D. Nugawela, Terence Stephenson, Roz Shafran, Bianca L. De Stavola, Shamez N. Ladhani, Ruth Simmons, Kelsey McOwat, Natalia Rojas, Emma Dalrymple, Emily Y. Cheung, Tamsin Ford, Isobel Heyman, Esther Crawley, Snehal M. Pinto Pereira

**Affiliations:** 1 UCL Great Ormond Street Institute of Child Health, London, UK; 2 Paediatric Infectious Diseases Research Group, St. George’s University of London, London, UK; 3 Immunisation Division, UK Health Security Agency, London, UK; 4 Department of Psychiatry, University of Cambridge, Cambridge, UK; 5 Centre for Academic Child Health, Bristol Medical School, University of Bristol, Bristol, UK; 6 Division of Surgery & Interventional Science, University College London, London, UK

**Keywords:** COVID-19, Long COVID, Symptoms, Predictive model, Public health, Children and young people

## Abstract

**Background:**

To update and internally validate a model to predict children and young people (CYP) most likely to experience long COVID (i.e. at least one impairing symptom) 3 months after SARS-CoV-2 PCR testing and to determine whether the impact of predictors differed by SARS-CoV-2 status.

**Methods:**

Data from a nationally matched cohort of SARS-CoV-2 test-positive and test-negative CYP aged 11–17 years was used. The main outcome measure, long COVID, was defined as one or more impairing symptoms 3 months after PCR testing. Potential pre-specified predictors included SARS-CoV-2 status, sex, age, ethnicity, deprivation, quality of life/functioning (five EQ-5D-Y items), physical and mental health and loneliness (prior to testing) and number of symptoms at testing. The model was developed using logistic regression; performance was assessed using calibration and discrimination measures; internal validation was performed via bootstrapping and the final model was adjusted for overfitting.

**Results:**

A total of 7139 (3246 test-positives, 3893 test-negatives) completing a questionnaire 3 months post-test were included. 25.2% (817/3246) of SARS-CoV-2 PCR-positives and 18.5% (719/3893) of SARS-CoV-2 PCR-negatives had one or more impairing symptoms 3 months post-test. The final model contained SARS-CoV-2 status, number of symptoms at testing, sex, age, ethnicity, physical and mental health, loneliness and four EQ-5D-Y items before testing. Internal validation showed minimal overfitting with excellent calibration and discrimination measures (optimism-adjusted calibration slope: 0.96575; C-statistic: 0.83130).

**Conclusions:**

We updated a risk prediction equation to identify those most at risk of long COVID 3 months after a SARS-CoV-2 PCR test which could serve as a useful triage and management tool for CYP during the ongoing pandemic. External validation is required before large-scale implementation.

**Supplementary Information:**

The online version contains supplementary material available at 10.1186/s12916-022-02664-y.

## Background

Children and young people (CYP) testing positive for SARS-CoV-2 are usually asymptomatic or have a low symptom burden at the time of infection compared to adults [1, 2]. Recent studies on post-COVID sequelae (also known as ‘long COVID’), however, have shown some adults and children can have persistent symptoms for months after acute infection [3, 4]. A recent systematic review of persistent symptoms following SARS-CoV-2 infection found most reported persistent symptoms were no more common in SARS-CoV-2-positive than in SARS-CoV-2-negative CYP, with only small increases in cognitive difficulties, headache, loss of smell, sore throat and sore eyes [5]. Similar to the successful use of predictive models for cardiovascular disease, e.g. in the United Kingdom (UK) [6] and the United States (US) [7], predictive models can help identify CYP at highest risk of experiencing persistent symptoms and direct them towards relevant care. This is particularly important during the pandemic when health services are under increased pressure [8]. A systematic review identified over 100 diagnostic and prognostic models for SARS-CoV-2, mainly relating to acute outcomes, e.g. mortality, intensive care unit (ICU) admission and length of hospital stay [9]. With the exception of two studies, however, most were considered low quality due to non-representative selection of controls, inadequate exclusions, high risk of model overfitting and unclear reporting [9]. Based on predictive model quality assessment tools [10] and model development guidelines [11], the two models mentioned above (the Jehi diagnostic model [12] and 4C mortality score [13]) and a third model (QCOVID [14]) are considered as higher quality predictive models for SARS-CoV-2 because of large sample sizes [15], appropriate modelling techniques [16] and suitable internal validation and reporting [11]. Of these three models, the 4C and QCOVID models were developed in adult populations (age≥18 years) whereas the Jehi model was developed in all patients who were tested for SARS-CoV-2 at all Cleveland Clinic locations in Ohio and Florida, US, regardless of age and included 11,672 patients (median age: 46.89 years among SARS-CoV-2 negatives; 54.23 years among SARS-CoV-2 positives).

There are very few predictive models for the potential long-term effects of SARS-CoV-2 infection, and those that exist have focused mostly on adults. Sudre and colleagues focused on identifying the characteristics and predictors of post-COVID sequelae in a sample of 4182 adults who reported testing positive for SARS-CoV-2 and found those experiencing more than five symptoms during the first week of illness were more likely to report ‘long COVID’ [17]. Recent large national cohort studies of CYP are consistent with the abovementioned systematic review [5], finding little difference in ‘long COVID’ symptom prevalence between SARS-CoV-2-positive and SARS-CoV-2 control CYP who either tested negative or did not have a test [4, 18]. As acute SARS-CoV-2 infection remains predominantly a mild infection in CYP and the cumulative incidence of infection increases, the incidence of post-COVID sequelae and the extent to which it is distinct from pandemic-related symptoms resulting from national lockdowns, school closures and social isolation is a critical factor in health policy decisions. We previously presented a model that predicted impairing symptoms in CYP [19], and here we aimed to update and internally validate the prediction model in CYP 3 months after a PCR test and to determine whether the impact of these predictors differed by SARS-CoV-2 infection status. The outcome examined here aligns with our previously described Delphi definition of long COVID [20].

## Methods

We use data from the Children and young people with Long Covid (CLoCk) study: a national cohort study of SARS-CoV-2 PCR-positive CYP aged 11–17 years living in England who were matched at study invitation, on month of test, age, sex and geographical area, to SARS-CoV-2 test-negative CYP selected from the national testing database at Public Health England (now UK Health Security Agency (UKHSA)) [21]. Test-negative CYP who self-reported subsequently testing positive for SARS-CoV-2 were excluded [4].

Here we examine a previously described study subset that is broadly representative of the target population in terms of age, sex, geographical region and socio-economic status [4]. Briefly, from a total of 50,836 CYP who were approached, 7139 (3246 SARS-CoV-2 positive, 3893 SARS-CoV-2 negative) who completed the CLoCk questionnaire sent to them 3 months after their PCR test during January–March 2021 (median time between testing and questionnaire: 14.9 weeks [25th, 50th centiles: 13.1, 18.9]) were included. The questionnaire included demographic characteristics, elements of the International Severe Acute Respiratory and emerging Infection Consortium (ISARIC) Paediatric COVID-19 follow-up questionnaire [22] and the recent Mental Health of Children and Young people in England surveys [23]. CYP responded to 21 questions on physical symptoms at the time of testing (e.g. cough, tiredness, etc.). They rated their general physical and mental health before SARS-CoV-2 testing in two separate questions using a 5-category Likert scale. The prevalence of ‘very poor’ was low; therefore, for analysis, we recoded these variables into four categories (very poor/poor to very good). Quality of life/functioning before testing was measured via the EQ-5D-Y scale [24], and feelings of loneliness by the UCLA Loneliness scale [25]. The Index of Multiple Deprivation (IMD) was calculated from the CYP’s small local area level-based geographic hierarchy (lower super output area) at the time of the questionnaire and used as a proxy for socio-economic status. We examine IMD quintiles from most (quintile 1) to least (quintile 5) deprived (Table 1).

**Table 1:** Baseline characteristics (frequencies and percentages) of participants who completed the 3-month questionnaire, overall and stratified by SARS-CoV-2 status

Characteristics at/prior to PCR testing	Total population (*N*=7139)		SARS-CoV-2 negative (*N*=3893)		SARS-CoV-2 positive (*N*=3246)	
	*N*	%	*N*	%	*N*	%
**Sex**						
Male	2646	37.06	1444	37.09	1202	37.03
Female	4493	62.94	2449	62.91	2044	62.97
**Age (years)**						
11–13	2212	30.98	1255	32.24	957	29.47
14–15	1883	26.38	997	25.61	886	27.30
16–17	3044	42.64	1641	42.15	1404	43.22
**Index of Multiple Deprivation**						
Quintile 1 (most deprived)	1471	20.61	785	20.16	686	21.13
Quintile 2	1422	19.92	761	19.55	661	20.36
Quintile 3	1367	19.15	764	19.62	603	18.58
Quintile 4	1377	19.29	752	19.32	625	19.25
Quintile 5 (least deprived)	1502	21.04	831	21.35	671	20.67
**Ethnicity**						
White	5279	73.95	2908	74.70	2371	73.04
Asian/Asian British	1064	14.90	550	14.13	514	15.83
Black/African/Caribbean	267	3.74	151	3.88	116	3.57
Mixed	358	45.01	203	5.21	155	4.78
Others	118	1.65	55	1.41	63	1.94
Preferred not to say	53	0.74	26	0.67	27	0.83
**Self-rated physical health**						
Very good	2443	34.22	1326	34.06	1117	34.41
Good	3017	42.26	1653	42.46	1364	42.02
Okay	1526	21.38	828	21.27	698	21.50
Poor/very poor	153	2.14	86	2.21	67	2.06
**Self-rated mental health**						
Very good	1789	25.06	960	24.66	829	25.54
Good	2634	36.90	1453	37.32	1181	36.38
Okay	2053	28.76	1109	28.49	944	29.08
Poor/very poor	663	9.29	371	9.53	292	9.00
**Loneliness**^**a**^						
Never	2079	29.12	1113	28.59	966	29.76
Hardly ever	2092	29.30	1119	28.74	973	29.98
Occasionally	1249	17.50	696	17.88	553	17.04
Some of the time	1229	17.22	675	17.34	554	17.07
Often/always	490	6.86	290	7.45	200	6.16
**Number of symptoms at testing**						
0	5673	79.46	3564	91.55	2109	64.97
1–4	556	7.79	178	4.58	378	11.65
5+	910	12.75	151	3.88	759	23.38
**Mobility**^**b**^						
No problems	6800	95.25	3694	94.89	3106	95.69
Some/a lot of problems	339	4.75	199	5.11	140	4.31
**Looking after self**^**b**^						
No problems	6837	95.77	3714	95.40	3123	96.21
Some/a lot of problems	302	4.23	179	4.60	123	3.79
**Doing usual activities**^**b**^						
No problems	6382	89.40	3487	89.57	2895	89.19
Some/a lot of problems	757	10.60	406	10.43	351	10.81
**Having pain**^**b**^						
No problems	6118	85.70	3342	85.85	2776	85.52
Some/a lot of problems	1021	14.30	551	14.15	470	14.48
**Feeling worried/sad**^**b**^						
No problems	4261	59.69	2331	59.88	1930	59.46
A bit	2421	33.91	1291	33.16	1130	34.81
Very worried/sad	457	6.40	271	6.96	186	5.73

^a^From the UCLA Loneliness scale

^b^From the EQ-5D-Y scale

### Outcome: long COVID (experiencing at least one impairing symptom)

We operationalized the Delphi research definition of long COVID [20] as having at least one of the 21 reported physical symptoms and experiencing more than minimal problems on any one of the five EQ-5D-Y questions at the time of the questionnaire, i.e. approximately 3 months after the PCR test (see Table 2). The published Delphi research definition requires laboratory confirmation of SARS-CoV-2 infection but of course that was not required when assessing how many test-negatives would also have met this definition.

**Table 2:** Prevalence (frequencies and percentages) of long COVID 3 months after a PCR test and related variables^a^, overall and stratified by SARS-CoV-2 status

Characteristics 3 months after a PCR test	Total population (*N*=7139)		SARS-CoV-2 negative (*N*=3893)		SARS-CoV-2 positive (*N*=3246)	
	*N*	%	*N*	%	*N*	%
**Outcome**						
**Long COVID at 3 months**						
No	5603	78.48	3174	81.53	2429	74.83
Yes	1536	21.52	719	18.47	817	25.17
**Variables related to the outcome**^**a**^						
**Number of symptoms**						
0	2968	41.57	1848	47.47	1120	34.50
1–4	3496	48.97	1798	46.19	1698	52.31
5+	675	9.46	247	6.34	428	13.19
**Mobility**						
No problems	6683	93.61	3680	94.53	3003	92.51
Some/a lot of problems	456	6.39	213	5.47	243	7.49
**Looking after self**						
No problems	6819	95.52	3709	95.27	3110	95.81
Some/a lot of problems	320	4.48	184	4.73	136	4.19
**Doing usual activities**						
No problems	6099	85.43	3376	86.72	2723	83.89
Some/a lot of problems	1040	14.57	517	13.28	523	16.11
**Having pain**						
No problems	6016	84.27	3327	85.46	2689	82.84
Some/a lot of problems	1123	15.73	566	14.54	557	17.16
**Feeling worried/sad**						
No problems/a bit	6571	92.04	3581	91.99	2990	92.11
Very worried/sad	568	7.96	312	8.01	256	7.89

The need for a positive test result was not required when assessing how many test-negatives would also have met this definition

^a^Using data from the questionnaire on the 21 symptoms and the EQ-5D-Y scale (~3 months after the PCR test), the Delphi research definition of long COVID was operationalized as having at least 1 symptom and experiencing some/a lot of problems with respect to mobility, self-care, doing usual activities or having pain/discomfort or feeling very worried/sad

### Potential predictors

Pre-specified potential predictors were chosen based on their distribution in the dataset and their association with the outcome. In addition to SARS-CoV-2 status, we considered 13 predictors including demographics (sex, age, ethnicity and IMD), prior quality of life/functioning (assessed by 5 items from the EQ-5D-Y scale), prior physical and mental health and feelings of loneliness prior to the CYP’s PCR test. We also included the number of physical symptoms experienced at testing (details in Table 1).

### Sample size and missing data

The sample size was pre-defined by the study design. We, therefore, assessed whether our study was sufficiently powered to estimate the overall outcome risk, and how many predictor parameters could be considered before overfitting/precision becomes a concern [15]. Using the pmsampsize STATA package [15], we considered (i) small overfitting (i.e. a shrinkage factor of predictor effects ≤10%), (ii) small absolute difference of 0.05 in the model’s apparent and adjusted Nagelkerke’s *R*-squared value and (iii) precise estimation within ±0.05 of the average outcome risk in the population. We also assumed an outcome prevalence of 21.5%, C-statistic of 0.80 and 61 parameters. Accordingly, the minimum sample size required was 2557 (actual sample=7139); the events-per-candidate predictor parameter value was 9.01. The dataset had no missing data.

### Statistical analysis

We assessed the extent to which SARS-CoV-2 status and our 13 potential predictors were correlated by considering pairwise Cramer’s *V* correlation coefficients. All potential predictors were categorical variables, with the exception of age and number of symptoms at testing. We determined the appropriate functional form for the relationship between age and the log odds of the probability of the outcome by modelling the relationship (i) linearly, (ii) categorically (11–13, 14–15, 16–17 years), (iii) with linear and quadratic terms and (iv) using fractional polynomials with up to 2 degrees. Similarly, we examined the most appropriate functional form for the number of symptoms. The functional form with the lowest Akaike information criterion (i.e., the best fitting model) was used in building our prediction model.

We used logistic regression to address our aim of predicting long COVID in CYP 3 months after their PCR test, allowing for an interaction between each potential predictor and SARS-CoV-2 status to determine whether the relationship between the potential predictor and outcome differed by SARS-CoV-2 status. We first examined univariable associations between each predictor and long COVID, in the total population and stratified by SARS-CoV-2 status. Next, we built a multivariable prediction model using a stepwise backward (*p*<0.200) and forward (*p*<0.157) elimination procedure [26]. Variables included in the stepwise selection procedure included all potential predictors, SARS-CoV-2 status and interaction terms between potential predictors and SARS-CoV-2 status (61 potential parameters in total). The above steps were used in developing our initial model for predicting long COVID [19], and here we present an update using a larger sample size and a refined definition of long COVID (see Table 2). The model was updated with an adjustment of the intercept to account for the difference in the outcome prevalence and all the regression coefficients were re-estimated based on the larger sample size of 7139 CYP.

Model performance was measured using calibration and discrimination measures. Calibration (i.e. agreement between observed and predicted probabilities of our outcome) was assessed using calibration plots, calibration-in-the-large and calibration slope statistics [16, 27]. Model discrimination (i.e. the ability of our model to differentiate between CYP who had long COVID 3 months post-test and those who did not) was quantified using the C-statistic (values ≥ 0.7 indicate strong discrimination). The internal validity of our final model was assessed using 100 bootstrap samples which were drawn with replacement [16]. We estimated the level of model overfitting (optimism) in our dataset using the bootstrap samples and adjusted for optimism using a uniform shrinkage factor (the average calibration slope from each of the bootstrap samples). The original *β* coefficients were multiplied by the shrinkage factor to obtain the optimism-adjusted coefficients; the model intercept was re-estimated based on these shrunken model coefficients generating the final model [11, 27].

Data management and analysis were performed using STATA16. We followed guidelines by the Prognosis Research Strategy (PROGRESS) [28–31] Group; the model development and validation phases particularly followed the suggested methods [27, 30–32]. The study is reported according to the Transparent Reporting of a multivariable prediction model for Individual Prognosis or Diagnosis (TRIPOD) statement (Additional file 1: Table 1) [11]. The study was approved by Yorkshire and the Humber–South Yorkshire Research Ethics Committee (REC reference: 21/YH/0060).

## Results

Of the 7139 CYP (3893 SARS-CoV-2 negative, 3246 SARS-CoV-2 positive) in our analytic sample, 26% (1860/7139) were of non-European origin, 62.9% (4493/7139) were female and there were more older than younger CYP (42.6% 16–17-year-olds vs. 31.0% 11–13-year-olds) (Table 1). Three months after their PCR test, 65.5% (2126/3246) of SARS-CoV-2 PCR-positives had at least one physical symptom (Table 2) and 25.2% (817/3246) had long COVID (i.e. at least one impairing symptom). This compares with 52.5% (2045/3893) and 18.5% (719/3893), respectively, in test-negative CYP.

### Univariable associations

SARS-CoV-2 status and the 13 potential predictors were not strongly correlated (Cramer’s *V* < 0.50 for all possible pairwise correlations). Ethnicity did not predict the outcome (Table 3). The predictive effect of self-rated physical and mental health, feelings of loneliness, problems with mobility, doing usual activities, having pain and feeling worried/sad before testing differed by SARS-CoV-2 status, with a general pattern of higher odds among test-negatives (Table 3, stratified associations).

**Table 3:** Odds ratios (95% CIs) of univariable associations between potential predictors and long COVID 3 months after a PCR test, overall and stratified by SARS-CoV-2 status

Potential predictor	Total population (*N*=7139)		SARS-CoV-2 negative (*N*=3893)		SARS-CoV-2 positive (*N*=3246)		*P*_interaction_^a^
	Odds ratio (95% CIs)	*p* value	Odds ratio (95% CIs)	*p* value	Odds ratio (95% CIs)	*p* value	
**SARS-CoV-2 status**							
Negative	Ref	**<0.001**					
Positive	1.48 (1.33, 1.66)		N/A		N/A		N/A
**Sex**							
Male	Ref	**<0.001**	Ref	**<0.001**	Ref	**<0.001**	0.5858
Female	2.02 (1.78, 2.30)		2.11 (1.75, 2.54)		1.96 (1.65, 2.34)		
**Age (years)**^**b**^							
11–13	Ref	**<0.001**	Ref	**<0.001**	Ref	**<0.001**	0.2533
14–15	1.54 (1.32, 1.80)		1.71 (1.37, 2.13)		1.36 (1.09, 1.70)		
16–17	1.73 (1.50, 1.99)		1.73 (1.42, 2.12)		1.70 (1.40, 2.07)		
**Index of Multiple Deprivation**							
Quintile 1 (most deprived)	Ref	**<0.001**	Ref	**0.034**	Ref	**0.004**	0.149
Quintile 2	0.99 (0.83, 1.17)		0.82 (0.64, 1.05)		1.18 (0.93, 1.49)		
Quintile 3	0.88 (0.74, 1.05)		0.79 (0.62, 1.02)		1.00 (0.78, 1.28)		
Quintile 4	0.79 (0.66, 0.94)		0.69 (0.53, 0.89)		0.90 (0.70, 1.16)		
Quintile 5 (least deprived)	0.72 (0.60, 0.86)		0.72 (0.56, 0.93)		0.72 (0.56, 0.93)		
**Ethnicity**							
White	Ref	0.055	Ref	0.146	Ref	0.131	0.417
Asian/Asian British	0.93 (0.79, 1.09)		0.93 (0.73, 1.18)		0.90 (0.72, 1.13)		
Black/African/Caribbean	1.11 (0.83, 1.48)		1.36 (0.92, 2.01)		0.89 (0.57, 1.38)		
Mixed	1.38 (1.09, 1.76)		1.43 (1.03, 2.00)		1.36 (0.95, 1.93)		
Others	0.70 (0.43, 1.16)		1.00 (0.50, 2.00)		0.49 (0.24, 1.00)		
Preferred not to say	0.96 (0.49, 1.87)		0.59 (0.18, 1.97)		1.23 (0.54, 2.83)		
**Self-rated physical health**							
Very good	Ref	**<0.001**	Ref	**<0.001**	Ref	**<0.001**	**<0.001**
Good	2.06 (1.77, 2.39)		2.49 (1.98, 3.13)		1.80 (1.47, 2.20)		
Okay	3.57 (3.04, 4.20)		4.51 (3.54, 5.76)		3.00 (2.40, 3.74)		
Poor/very poor	7.60 (5.41, 10.68)		14.91 (9.32, 23.85)		3.71 (2.23, 6.19)		
**Self-rated mental health**							
Very good	Ref	**<0.001**	Ref	**<0.001**	Ref	**<0.001**	**0.015**
Good	2.12 (1.75, 2.56)		2.55 (1.87, 3.81)		1.92 (1.50, 2.46)		
Okay	3.91 (3.23, 4.73)		5.50 (4.06, 7.46)		3.06 (2.39, 3.93)		
Poor/very poor	12.72 (10.17, 15,91)		17.46 (12.43, 24.53)		10.54 (7.72, 14.40)		
**Loneliness**^**c**^							
Never	Ref	**<0.001**	Ref	**<0.001**	Ref	**<0.001**	**0.002**
Hardly ever	1.75 (1.45, 2.11)		2.16 (1.59, 2.93)		1.54 (1.21, 1.96)		
Occasionally	3.91 (3.23, 4.73)		5.28 (3.91, 7.14)		3.30 (2.56, 4.26)		
Some of the time	4.81 (3.98, 5.80)		6.86 (5.10, 9.22)		3.82 (2.97, 4.91)		
Often/always	7.98 (6.34, 10.04)		13.41 (9.56, 18.80)		5.23 (3.75, 7.30)		
**Number of symptoms at testing**^**b**^							
0	Ref	**<0.001**	Ref	**<0.001**	Ref	**<0.001**	0.148
1–4	0.43 (0.32, 0.57)		0.41 (0.24, 0.70)		0.37 (0.26, 0.52)		
5+	1.88 (1.62, 2.19)		2.20 (1.55, 3.12)		1.49 (1.24, 1.79)		
**Mobility**^**d**^							
No problems	Ref	**<0.001**	Ref	**<0.001**	Ref	**<0.001**	**0.024**
Some/a lot of problems	6.81 (5.42, 8.55)		8.82 (6.53, 11.91)		5.17 (3.65, 7.34)		
**Looking after self**^**d**^							
No problems	Ref	**<0.001**	Ref	**<0.001**	Ref	**<0.001**	0.205
Some/a lot of problems	8.89 (6.92, 11.40)		10.58 (7.65, 14.62)		7.61 (5.13, 11.28)		
**Doing usual activities**^**d**^							
No problems	Ref	**<0.001**	Ref	**<0.001**	Ref	**<0.001**	**< 0.001**
Some/a lot of problems	6.52 (5.57, 7.64)		10.31 (8.26, 12.88)		4.04 (3.22, 5.08)		
**Having pain**^**d**^							
No problems	Ref	**<0.001**	Ref	**<0.001**	Ref	**<0.001**	**<0.001**
Some/a lot of problems	9.84 (8.51, 11.38)		14.66 (11.94, 18.00)		6.65 (5.40, 8.18)		
**Feeling worried/sad**^**d**^							
No problems	Ref	**<0.001**	Ref	**<0.001**	Ref	**<0.001**	**0.006**
A bit	4.05 (3.56, 4.60)		5.11 (4.21, 6.21)		3.35 (2.81, 3.99)		
Very worried/sad	15.12 (12.20, 18.75)		17.54 (13.17, 23.36)		15.38 (10.90, 21.70)		

^a^*P* value for interaction between potential predictor and SARS-CoV-2 status (derived in total population)

^b^We show (for simplicity) univariable associations for age and number of symptoms modelled as categorical variables. In the final multivariable model, they have been modelled with linear and quadratic terms or a fractional polynomial; see the “Methods” section for details

^c^From the UCLA Loneliness scale. ^d^From the EQ-5D-Y scale

### Multivariable predictive model

In the final model (Additional file 1: Table 2), SARS-CoV-2 status, number of symptoms at testing, sex, age, ethnicity, self-rated physical and mental health, feelings of loneliness and four items from the EQ-5D-Y scale (problems looking after self, doing usual activities, having pain, feeling worried/sad) before testing predicted the outcome. The impact of some predictors differed by SARS-CoV-2 status: interactions between SARS-CoV-2 status and age, ethnicity, self-rated mental health, feelings of loneliness and problems doing usual activities were retained. Additional file 1: Fig. 1 shows graphs from the final model, for all included predictors, of the probability of having the outcome.

### Model performance

**Fig. 1:**
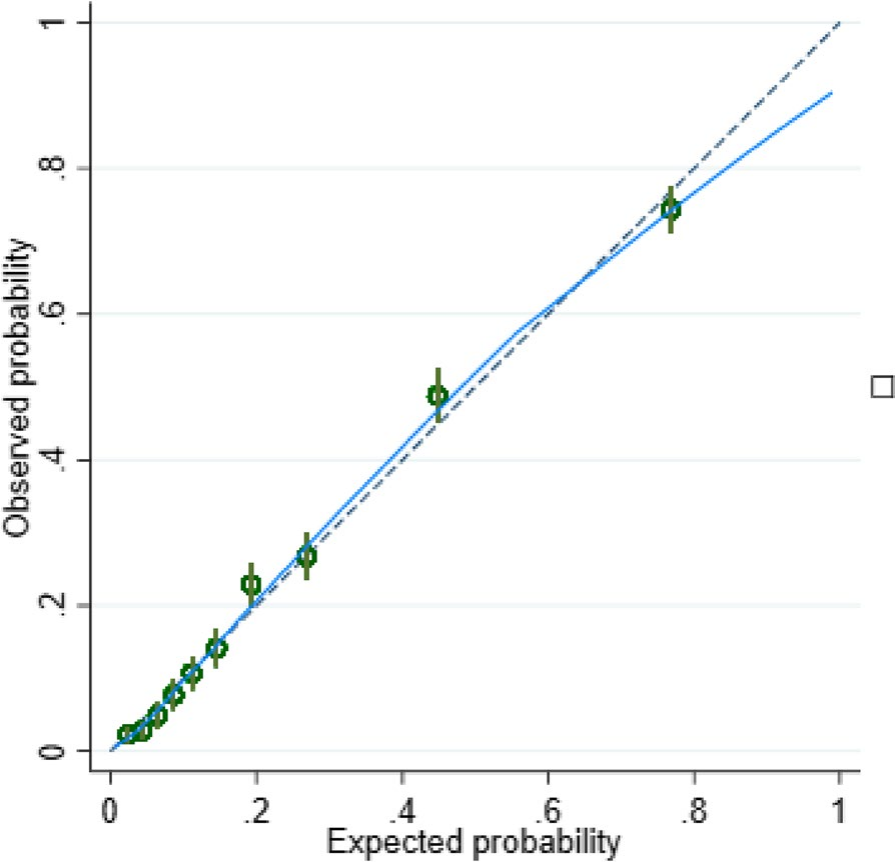
Observed and predicted risk of long COVID 3 months after a PCR test. This graph shows the mean predicted probability (hollow dots) and 95% confidence intervals of long COVID 3 months after a PCR test plotted against the observed proportion of the same outcome for 10 equally sized groups. The dashed line represents the line of equality and perfect calibration. The blue solid line is a smoothed locally weighted scatter plot smoothing (Lowess) regression line

The model showed excellent calibration and discrimination. It was perfectly calibrated in the model development data with an apparent slope of 1 and an apparent calibration-in-the-large of 0 (Additional file 1: Table 3). Good overall model calibration was further confirmed by the calibration plot (Fig. 1), with narrow confidence intervals and closely aligned predicted and observed probabilities for 10 equally sized risk groups. The predictive model showed strong discrimination with a C-statistic of 0.838 (95% CI: 0.827, 0.849). Bootstrap internal validation showed small model overfitting with an optimism-corrected C slope close to one. The bootstrapping approach provided a shrinkage factor of 0.965752; we also generated the heuristic shrinkage factor (again close to one: 0.979196). We chose the bootstrap shrinkage factor as it was slightly smaller, and applied it to the original *β* coefficients to obtain the optimism-adjusted coefficients before re-estimating the intercept for the final model given in Box 1 (Additional file 1) and Additional file 1: Table 2.

### Worked examples

Box 1 (Additional file 1) shows the prediction equation for estimating the risk of long COVID 3 months post-PCR test in 11-to-17-year-old CYP. We demonstrate with hypothetical examples the predicted risk of long COVID 3 months post-test in Table 4. A calculator is provided in Additional file 2.

**Table 4:** Hypothetical examples of predicted risk of long COVID 3 months after a PCR test, using our prediction model

Characteristic	Examples					
	1	2	3	4	5	6
SARS-CoV-2 status	Positive	Negative	Positive	Negative	Positive	Negative
Sex	Female	Female	Male	Male	Female	Female
Age (years)	17	17	13	13	17	17
Ethnicity	White	White	White	White	White	White
Self-rated physical health	Okay	Okay	Okay	Okay	Good	Good
Self-rated mental health	Okay	Okay	Okay	Okay	Good	Good
Loneliness	Some of the time	Some of the time	Some of the time	Some of the time	Hardly ever	Hardly ever
Number of symptoms at time of testing	0	0	0	0	3	3
Problems looking after myself	No problems	No problems	No problems	No problems	No problems	No problems
Problems doing usual activities	No problems	No problems	No problems	No problems	No problems	No problems
Having pain	No problems	No problems	No problems	No problems	No problems	No problems
Feeling worried/sad	A bit worried	A bit worried	A bit worried	A bit worried	No problems	No problems
**Predicted risk of long COVID**	**0.342**	**0.217**	**0.241**	**0.189**	**0.076**	**0.030**

For details on how the predicted risk was calculated, see the supplementary section for the formula (Box 1) and calculator

As an example, the predicted risk of outcome for a hypothetical 14-year-old, white male, with no symptoms at testing, very good physical health, never feeling lonely, no problems on all included EQ-5D-Y items and poor/very poor mental health before testing, would be 0.11 if he tested positive and 0.04 if negative; if he had very good mental health before testing, the risk would be 0.07 if positive and 0.03 if negative.

## Discussion

To our knowledge, we have developed [19] and updated the first risk prediction model that uses self-reported information from CYP to estimate their probability of experiencing long COVID 3 months after SARS-CoV-2 testing. SARS-CoV-2 status, number of physical symptoms at testing, sex, age, ethnicity, self-rated physical and mental health, feelings of loneliness and four items from the EQ-5D-Y scale (all before testing) predicted long COVID 3 months later, with the impact of some predictors differing by SARS-CoV-2 status. We provide a risk calculator to predict CYP most likely to experience long COVID, to triage those who need support and for whom early intervention might be of greatest benefit. Importantly, our model has excellent predictive ability, calibration and discrimination. It enables us to answer important clinical questions such as ‘are those who have many symptoms during acute SARS-CoV-2 infection at greater risk of Long COVID than those without?’. The answer is ‘yes’ but our model provides a more nuanced answer by considering other factors.

Our goal was to provide a model that utilizes multiple factors (i.e. predictors) in combination, to accurately predict long COVID 3 months post-test. Importantly, our focus was not on whether included predictors are causal or not. Instead, the focus was the overall predictive performance of the model [33]. As such, we followed the guidelines to model building [27]. The large sample allowed flexible examination of the potential for relationships to differ by SARS-CoV-2 status and by the shape of the association without considerable concerns about overfitting. Model fitting statistics were extremely favourable and the use of a matched national cohort sample of test-positive and test-negative CYP is unique. Despite its internal validation, the model needs to be externally validated on other independent datasets and in different populations and settings prior to its wider application. Additionally, the model needs to be reassessed for experiencing long COVID beyond 3 months. It is possible many of the predictors stay the same but acknowledge there may be differences as the disease profile (and, therefore, predictors) changes over the course of the illness.

We acknowledge study limitations. The CLoCk study response rate (13.9%) is typical of surveys of this type [34] and is in line with other COVID-19-related studies [35, 36]. Importantly, the examined CYP are broadly representative of the target population in terms of important demographics such as age, sex and socio-economic status [4] as well as more generally of CYP aged 11–17 years living in England [37]. Baseline measures (at/or before testing) were subject to recall bias because they were not taken at the time of acute infection, and we were unable to assess whether symptoms waxed and waned between testing and the questionnaire. In addition, the possibility of selection bias in both directions (CYP more likely to participate if they have persistent symptoms, or less likely to participate if too unwell) among respondents cannot be ruled out. Furthermore, as the background epidemiological situation in relation to SARS-CoV-2 infection prevalence changes, there is a need to reassess possible differences in our model’s predictive value over time. Finally, caution is required for predictions based on data extrapolation/situations where there are only a very small number of observations for different predictor combinations.

To our knowledge, no other study has explicitly aimed to present a risk prediction model for long COVID [5, 38]. Moreover, the majority of previous studies lack a SARS-CoV-2 test-negative comparison group and so distinguishing long-term symptoms predicted by SARS-CoV-2 infection from background rates or pandemic-related effects remains a challenge [5]. More recent studies include control groups and, thus, broad comparisons can be made. Our finding that the odds of experiencing long COVID 3 months post-test was 1.48 times higher in SARS-CoV-2-positive compared to SARS-CoV-2-negative CYP is in line with findings from the LongCOVIDKidsDK study, where the SARS-CoV-2 test-positive group had 1.22 times higher odds of having at least one ‘Long COVID’ symptom lasting at least 2 months compared with the SARS-CoV-2 control group who either tested negative or never had a test [39]. We found both test-positive and test-negative CYP met the Delphi consensus definition of long COVID 3 months post-test with a difference of 6.7% between these groups. In contrast, in Borch et al., the prevalence of reported symptoms in CYP aged 6–17 years lasting more than 4 weeks was similar regardless of SARS-CoV-2 status (28% test-positives; 27.2% test-negatives/never had a test) [18]. Discrepancies in findings could be due to several reasons including differences in the symptom questions asked of the test-positive and test-negative/never been tested groups, timing of outcome (>4 weeks vs ~3 months), recruitment methodology, recruitment rates between test-positives and test-negatives and/or underlying prevalence levels in the countries at the time of the study. Our results are consistent with findings in adults, where the number of symptoms at onset [40] and female sex [41] were associated with ‘Long COVID’ and pre-existing diagnosis of depression/anxiety is over-represented in those with fatigue after SARS-CoV-2 infection [41].

## Conclusions

Understanding which CYP are at risk of experiencing long COVID is important for individuals (e.g. in decision-making about whether to receive COVID-19 vaccination) and health service provision (e.g. for careful monitoring, early intervention and hopefully reduction in the burden of prolonged health problems). Using data from a large national matched cohort study, we updated our previously developed prediction model for experiencing long COVID 3 months after SARS-CoV-2 testing in CYP. Our model has excellent performance, and we hope it will serve as a useful tool for the early identification and management of CYP at risk of long COVID in the context of the current pandemic.

## Supplementary Information

**Additional file 1: Table 1.** TRIPOD checklist for prognostic model development and validation studies. **Table 2.** Final multivariable analysis developed model and optimism adjusted β coefficients. **Table 3.** Model Performance Statistics based on internal validation. **Figure 1.** Probability of long COVID for each predictor (from the developed model), when all other predictive variables are at their reference value. **Box 1.** Final equation for experiencing long COVID 3 months after a PCR-test in children aged 11 to 17 years.

**Additional file 2.** Risk Calculator for experiencing long COVID 3 months after a PCR test.

## Data Availability

Data are not publicly available. All requests for data will be reviewed by the Children & young people with Long Covid (CLoCk) study team, to verify whether the request is subject to any intellectual property or confidentiality obligations. Requests for access to the participant-level data from this study can be submitted via email to clock@phe.gov.uk with detailed proposals for approval. A signed data access agreement with the CLoCK team is required before accessing shared data. Code is not made available as we have not used custom code or algorithms central to our conclusions.

## Abbreviations

CLoCk: Children and young people with Long Covid
CYP: Children and young people
ICU: Intensive care unit
IMD: Index of Multiple Deprivation
ISARIC: International Severe Acute Respiratory and emerging Infection Consortium
UK: United Kingdom
UKHSA: UK Health Security Agency
US: United States
