# Supplementary material for: Predictive model for long COVID in children 3 months after a SARS-CoV-2 PCR test
Source: BMC Med. 2022 Nov 30;20:465. doi: 10.1186/s12916-022-02664-y (PMC9708506; doi:10.1186/s12916-022-02664-y)
Supplement: Supplementary file 1 — Additional file 1: Table 1. TRIPOD checklist for prognostic model development and validation studies. Table 2. Final multivariable analysis developed model and optimism adjusted β coefficients. Table 3. Model Performance Statistics based on internal validation. Figure 1. Probability of long COVID for each predictor (from the developed model), when all other predictive variables are at their reference value. Box 1. Final equation for experiencing long COVID 3 months after a PCR-test in children aged 11 to 17 years. [file 12916_2022_2664_MOESM1_ESM.docx]

**Additional File Table 1:** TRIPOD checklist for prognostic model development and validation studies

| **Section/Topic** | **Item** |  | **Checklist Item** | **Page** |
| --- | --- | --- | --- | --- |
| **Title and abstract** | | | | |
| Title | 1 | D;V | Identify the study as developing and/or validating a multivariable prediction model, the target population, and the outcome to be predicted. | 1 |
| Abstract | 2 | D;V | Provide a summary of objectives, study design, setting, participants, sample size, predictors, outcome, statistical analysis, results, and conclusions. | 2-3 |
| **Introduction** | | | | |
| Background and objectives | 3a | D;V | Explain the medical context (including whether diagnostic or prognostic) and rationale for developing or validating the multivariable prediction model, including references to existing models. | 4-5 |
|  | 3b | D;V | Specify the objectives, including whether the study describes the development or validation of the model or both. | 5 |
| **Methods** | | | | |
| Source of data | 4a | D;V | Describe the study design or source of data (e.g., randomized trial, cohort, or registry data), separately for the development and validation data sets, if applicable. | 6 |
|  | 4b | D;V | Specify the key study dates, including start of accrual; end of accrual; and, if applicable, end of follow-up. | 6 |
| Participants | 5a | D;V | Specify key elements of the study setting (e.g., primary care, secondary care, general population) including number and location of centres. | 6 |
|  | 5b | D;V | Describe eligibility criteria for participants. | 6 |
|  | 5c | D;V | Give details of treatments received, if relevant. | N/A |
| Outcome | 6a | D;V | Clearly define the outcome that is predicted by the prediction model, including how and when assessed. | 7 |
|  | 6b | D;V | Report any actions to blind assessment of the outcome to be predicted. | N/A |
| Predictors | 7a | D;V | Clearly define all predictors used in developing or validating the multivariable prediction model, including how and when they were measured. | 7-8 |
|  | 7b | D;V | Report any actions to blind assessment of predictors for the outcome and other predictors. | N/A |
| Sample size | 8 | D;V | Explain how the study size was arrived at. | 8 |
| Missing data | 9 | D;V | Describe how missing data were handled (e.g., complete-case analysis, single imputation, multiple imputation) with details of any imputation method. | 8 |
| Statistical analysis methods | 10a | D | Describe how predictors were handled in the analyses. | 8-9 |
|  | 10b | D | Specify type of model, all model-building procedures (including any predictor selection), and method for internal validation. | 9-10 |
|  | 10c | V | For validation, describe how the predictions were calculated. | 9-10 |
|  | 10d | D;V | Specify all measures used to assess model performance and, if relevant, to compare multiple models. | 9-10 |
|  | 10e | V | Describe any model updating (e.g., recalibration) arising from the validation, if done. | 9 |
| Risk groups | 11 | D;V | Provide details on how risk groups were created, if done. | N/A |
| Development vs. validation | 12 | V | For validation, identify any differences from the development data in setting, eligibility criteria, outcome, and predictors. | N/A |
| **Results** | | | | |
| Participants | 13a | D;V | Describe the flow of participants through the study, including the number of participants with and without the outcome and, if applicable, a summary of the follow-up time. A diagram may be helpful. | 10-11 |
|  | 13b | D;V | Describe the characteristics of the participants (basic demographics, clinical features, available predictors), including the number of participants with missing data for predictors and outcome. | 10-11 |
|  | 13c | V | For validation, show a comparison with the development data of the distribution of important variables (demographics, predictors and outcome). | N/A |
| Model development | 14a | D | Specify the number of participants and outcome events in each analysis. | 10-11 |
|  | 14b | D | If done, report the unadjusted association between each candidate predictor and outcome. | 11 |
| Model specification | 15a | D | Present the full prediction model to allow predictions for individuals (i.e., all regression coefficients, and model intercept or baseline survival at a given time point). | Supplement |
|  | 15b | D | Explain how to the use the prediction model. | 12 |
| Model performance | 16 | D;V | Report performance measures (with CIs) for the prediction model. | 12 |
| Model-updating | 17 | V | If done, report the results from any model updating (i.e., model specification, model performance). | 11-12 |
| **Discussion** | | | | |
| Limitations | 18 | D;V | Discuss any limitations of the study (such as nonrepresentative sample, few events per predictor, missing data). | 14 |
| Interpretation | 19a | V | For validation, discuss the results with reference to performance in the development data, and any other validation data. | N/A |
|  | 19b | D;V | Give an overall interpretation of the results, considering objectives, limitations, results from similar studies, and other relevant evidence. | 13-15 |
| Implications | 20 | D;V | Discuss the potential clinical use of the model and implications for future research. | 15-16 |
| **Other information** | | | | |
| Supplementary information | 21 | D;V | Provide information about the availability of supplementary resources, such as study protocol, Web calculator, and data sets. | Supplement |
| Funding | 22 | D;V | Give the source of funding and the role of the funders for the present study. | 17 |

*Items relevant only to the development of a prediction model are denoted by D, items relating solely to a validation of a prediction model are denoted by V, and items relating to both are denoted D;V.

**Additional File Table 2:** Final multivariable analysis developed model and optimism adjusted β coefficients

| Variable | Developed model: coefficients | Final model coefficients after adjusting for overfitting |
| --- | --- | --- |
| **SARS-CoV-2 test result** |  |  |
| Negative | 0.00000 | 0.00000 |
| Positive | 1.03388 | 0.99847 |
| **Sex** |  |  |
| Male | 0.00000 | 0.00000 |
| Female | 0.36324 | 0.35080 |
| **Ethnicity** |  |  |
| White | 0.00000 | 0.00000 |
| Asian | 0.08161 | 0.07882 |
| Black | 0.25060 | 0.24203 |
| Mixed | 0.45300 | 0.43749 |
| Other | 0.13034 | 0.12588 |
| Prefer not to say | -0.58041 | -0.56053 |
| **Physical Health** **before testing** |  |  |
| Very good | 0.00000 | 0.00000 |
| Good | 0.31963 | 0.30868 |
| Okay | 0.44983 | 0.43443 |
| Very poor/Poor | 0.48992 | 0.47315 |
| **Mental Health before testing** |  |  |
| Very good | 0.00000 | 0.00000 |
| Good | 0.25949 | 0.25060 |
| Okay | 0.39083 | 0.37745 |
| Very poor/Poor | 0.45189 | 0.43641 |
| **Loneliness before testing** |  |  |
| Never | 0.00000 | 0.00000 |
| Hardly Ever | 0.46431 | 0.44841 |
| Occasionally | 0.83599 | 0.80736 |
| Some of the time | 0.86956 | 0.83978 |
| Often/Always | 0.98939 | 0.95551 |
| **Looking after self before testing** |  |  |
| No problem | 0.00000 | 0.00000 |
| Some/a lot of problems | 0.99648 | 0.96235 |
| **Doing usual activities before testing** |  |  |
| No problem | 0.00000 | 0.00000 |
| Some/a lot of problems | 0.78959 | 0.76255 |
| **Having pain before testing** |  |  |
| No problem | 0.00000 | 0.00000 |
| Some/a lot of problems | 1.73252 | 1.67319 |
| **Feeling worried/sad before testing** |  |  |
| No problem | 0.00000 | 0.00000 |
| A bit | 0.63393 | 0.61222 |
| Very worried/sad | 1.27242 | 1.22884 |
| **Age at time of testing**^#^ |  |  |
| (Age-14) | 0.02397 | 0.02315 |
| (Age-14)^2^ | -0.03524 | -0.03404 |
| **Number of symptoms at time of testing** |  |  |
| ((number of symptoms+1)/10)^-2^ | 0.01773 | 0.01712 |
| (number of symptoms+1) /10 | 2.32994 | 2.25015 |
| **Ethnicity*Positive SARS-CoV-2 test result** |  |  |
| White | 0.00000 | 0.00000 |
| Asian | -0.16281 | -0.15724 |
| Black | -0.48247 | -0.46595 |
| Mixed | -0.28892 | -0.27903 |
| Other | -0.89708 | -0.86636 |
| Prefer not to say | 1.27248 | 1.22891 |
| **Mental Health before testing*Positive SARS-CoV-2 test result** |  |  |
| Very good | 0.00000 | 0.00000 |
| Good | -0.12697 | -0.12262 |
| Okay | -0.40864 | -0.39465 |
| Very poor/Poor | 0.18387 | 0.17757 |
| **Loneliness before testing*Positive SARS-CoV-2 test result** |  |  |
| Never | 0.00000 | 0.00000 |
| Hardly Ever | -0.29911 | -0.28887 |
| Occasionally | -0.40995 | -0.39591 |
| Some of the time | -0.37543 | -0.36258 |
| Often/Always | -0.65366 | -0.63128 |
| **Doing usual activities before testing*Positive SARS-CoV-2 test result** |  |  |
| No problems | 0.00000 | 0.00000 |
| Some/a lot of problems | -0.90103 | -0.87018 |
| **Age*Positive SARS-CoV-2 test result** |  |  |
| **(Age-14)** | -0.01905 | -0.0184 |
| **(Age-14)^2^** | 0.05130 | 0.04955 |
| **Constant*** | -5.76129 | -5.6010 |

Outcome modelled is the ln-odds of long COVID 3 months after a PCR-test i.e. ln(P_i_/(1-P_i_)) where “P_i_” is the probability of long COVID 3 months after a PCR-test for person i and “ln” is natural logarithmic transformation

^#^Age was centered on 14 years i.e. (Age-14)

*Constant term was re-estimated after adjustment for optimism (shrinkage factor =0.96575) to uphold overall model calibration

**Additional File Table 3:** Model Performance Statistics based on internal validation

| Measure | Apparent performance (95% CI) | Average optimism | Optimism corrected |
| --- | --- | --- | --- |
| Calibration slope* | 1.00000 (0.99998, 1.00001) | 0.03425 | 0.96575 |
| Calibration in the large (CITL)** | 0.00000 (-0.00005, 0.00005) | -0.00620 | 0.00620 |
| C Statistic*** | 0.83788 (0.82672, 0.84903) | 0.00658 | 0.83130 |

*A measure of calibration; Values closer to one indicate better calibration.

**A measure of calibration; values closer to zero indicate better calibration

***A measure of discrimination; values 0.7 and above indicate strong discrimination

| **Box 1**: Final equation for experiencing long COVID 3 months after a PCR-test in children aged 11 to 17 years  **Estimated risk of experiencing long COVID 3 months after a PCR-test** = exp (Linear Predictor)/ (1+exp (Linear Predictor))  Where:  Linear Predictor = -5.60102 + 0.35080*[sex=Female] + 0.07881*[Ethnicity=Asian]*[SARS-CoV-2 test result=Negative]+ 0.24202*[Ethnicity=Black]*[SARS-CoV-2 test result=Negative] + 0.43749*[Ethnicity=Mixed]*[SARS-CoV-2 test result=Negative] +0.125878*[Ethnicity=Other]*[SARS-CoV-2 test result=Negative]- 0.56053*[Ethnicity=Prefer not to say]*[SARS-CoV-2 test result=Negative] + 0.47315*[Physical Health=Very poor/Poor] + 0.43443*[Physical Health=Okay] +0.30868*[Physical Health=Good] + 0.4364132*[Mental Health=Very poor/Poor]*[SARS-CoV-2 test result=Negative] + 0.37745*[Mental Health=Okay]*[SARS-CoV-2 test result=Negative] + 0.25060*[Mental Health=Good]*[SARS-CoV-2 test result=Negative] + 0.95551*[Loneliness=Often/Always]*[SARS-CoV-2 test result=Negative] + 0.83978*[Loneliness=Some of the time]*[SARS-CoV-2 test result=Negative]+ 0.80736*[Loneliness=Occasionally]*[SARS-CoV-2 test result=Negative] + 0.44841*[Loneliness=Hardly Ever]*[SARS-CoV-2 test result=Negative] + 0.962351*[EQ-5D-Y_look=Some/a lot of problems] + 0.76255*[EQ-5D-Y_usual=Some/a lot of problems]*[SARS-CoV-2 test result=Negative] + 1.67319*[EQ-5D-Y_pain=Some/a lot of problems] + 0.61222*[EQ-5D-Y_sad=A bit] + 1.22884*[EQ-5D-Y_sad=Very] + 0.02315*(Age-14)*[SARS-CoV-2 test result=Negative] + (-0.03404)*(Age-14)^2^ *[SARS-CoV-2 test result=Negative] -0.07842 *[Ethnicity=Asian]*[SARS-CoV-2 test result=Positive] -0.22392*[Ethnicity=Black]*[SARS-CoV-2 test result=Positive] 0.15846*[Ethnicity=Mixed]*[SARS-CoV-2 test result=Positive] -0.740487 *[Ethnicity=Other]*[SARS-CoV-2 test result=Positive] 0.66838*[Ethnicity=Prefer not to say]*[SARS-CoV-2 test result=Positive] + 0.61398*[Mental Health=Very poor/Poor]*[SARS-CoV-2 test result=Positive] -0.0172*[Mental Health=Okay]*[SARS-CoV-2 test result=1]+0.12798*[Mental Health=Good]*[SARS-CoV-2 test result=Positive] + 0.32422*[Loneliness=Often/Always]*[SARS-CoV-2 test result=Positive] + 0.47720*[Loneliness=Some of the time]*[SARS-CoV-2 test result=Positive] + 0.41145*[Loneliness=Occasionally]*[SARS-CoV-2 test result=Positive] + 0.15954*[Loneliness=Hardly Ever]*[SARS-CoV-2 test result=Positive] -0.10763*[EQ-5D-Y_usual=Some problems]*[SARS-CoV-2 test result=Positive] + 0.99847*[SARS-CoV-2 test result=Positive] + 0.00475*(Age-14)*[SARS-CoV-2 test result=Positive] + 0.01551*(Age-14)^2^ *[SARS-CoV-2 test result=Positive] + (0.01712)* ((Total number of symptoms+1)/10)^2^ + (2.25015)*((Total number of symptoms+1)/10)  exp = exponential function  **Note in above Linear Predictor:**   - score 1 if sex is female indicated as [sex=Female] - score 1 if ethnicity is Asian indicated as [Ethnicity=Asian]; similarly for Black, Mixed, Other, or Prefer not to say - score 1 if physical health before testing was good indicated as [Physical Health=Good]; similarly for okay, or very poor/poor - score 1 if mental health before testing was good indicated as [Mental Health=Good]; similarly for okay, or very poor/poor - score 1 if felt lonely often/always before testing indicated as [Loneliness=Often/Always]; similarly for some of the time, occasionally, hardly ever - score 1 if had some/a lot of problems looking after self before testing indicated as [EQ-5D-Y_look=Some/a lot of problems]; similarly for doing usual activities before testing (EQ-5D-Y_usual) and having pain before testing (EQ-5D-Y_pain) - score 1 if was a bit sad indicated as [EQ-5D-Y_sad=A bit]; similarly for very worried/sad   See Table 4 (main test) for worked examples. |
| --- |

**Additional File Figure 1**: Probability of long COVID for each predictor (from the developed model), when all other predictive variables are at their reference value*

1. Age (years) (b) Total symptoms


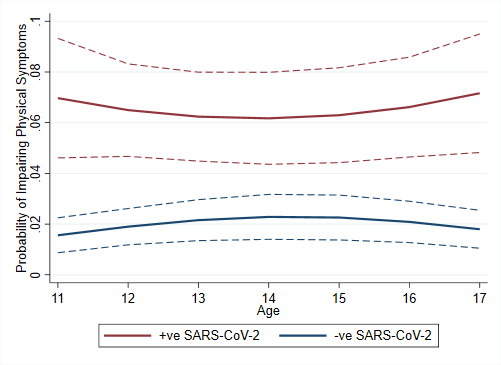

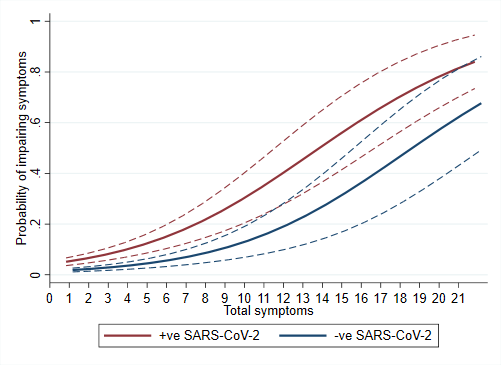


1. Sex (d) Ethnicity


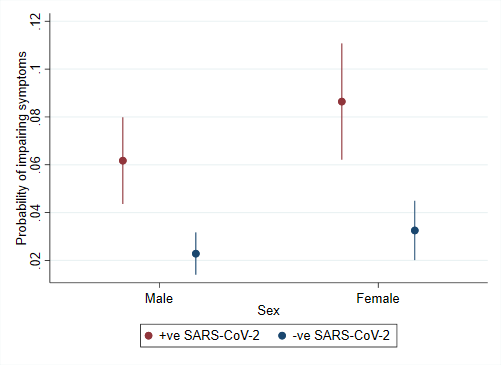

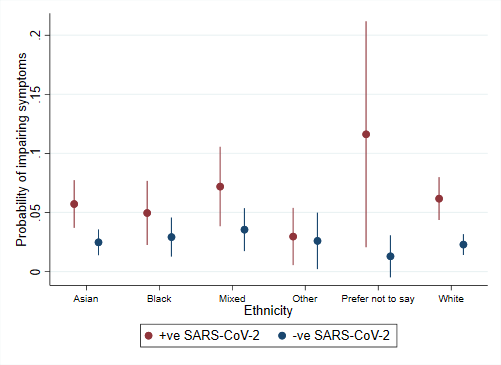


(e) Mental health (f) Physical Health


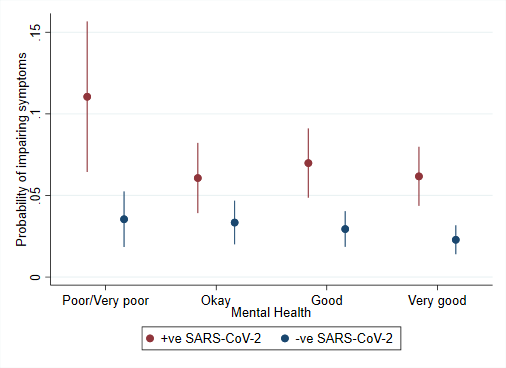

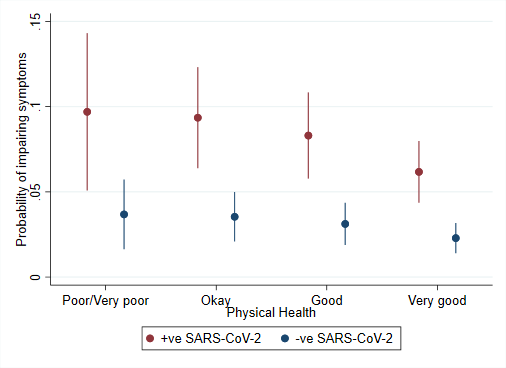


(g) Loneliness


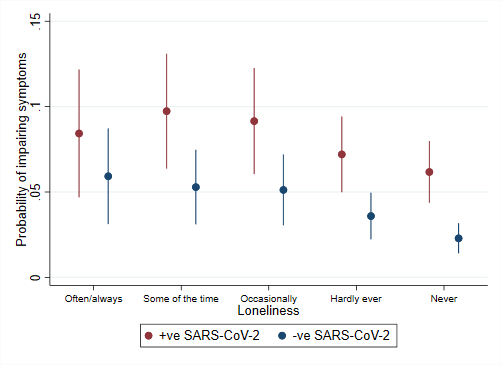


(h) EQ-5D-Y Looking after self (i) EQ-5D-Y Feeling sad/worried


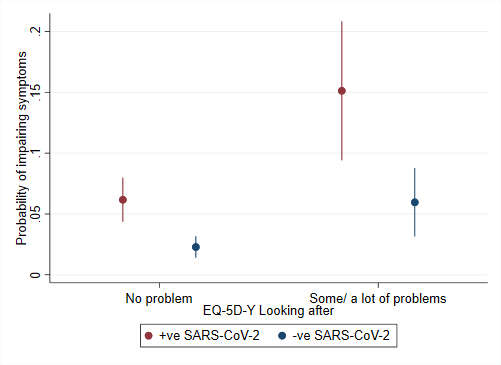

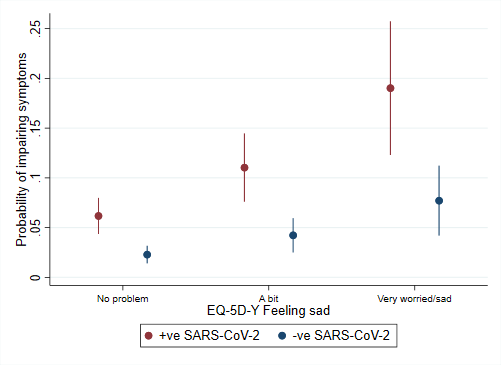


(j) EQ-5D-Y Having pain (k) EQ-5D-Y Doing usual activities


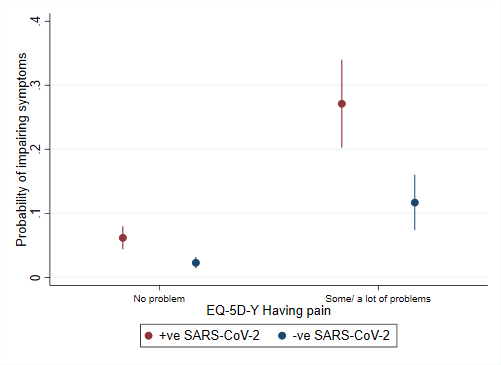

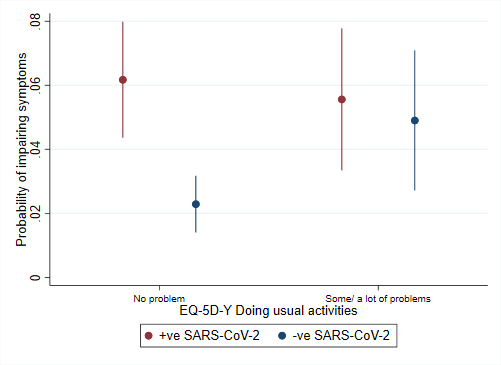


*Reference values are: 14 years, male, White ethnicity, zero symptoms, very good physical health, very good mental health, never feeling lonely and no problems on all included EQ-5D-Y items
